# What do randomized controlled trials say about virtual rehabilitation in stroke? A systematic literature review and meta-analysis of upper-limb and cognitive outcomes

**DOI:** 10.1186/s12984-018-0370-2

**Published:** 2018-03-27

**Authors:** Anna Aminov, Jeffrey M. Rogers, Sandy Middleton, Karen Caeyenberghs, Peter H. Wilson

**Affiliations:** 1 School of Psychology, Faculty of Health Sciences, Australian Catholic University, Sydney, NSW Australia; 2 South Eastern Sydney Local Health District, Sydney, NSW Australia; 3 School of Psychology, Australian Catholic University, Melbourne, VIC Australia; 4 Centre for Disability and Development Research (CeDDR), Australian Catholic University, Melbourne, VIC Australia

**Keywords:** Cognition, Meta-analysis, Motor performance, Rehabilitation, Stroke, Virtual reality

## Abstract

**Background:**

Virtual-reality based rehabilitation (VR) shows potential as an engaging and effective way to improve upper-limb function and cognitive abilities following a stroke. However, an updated synthesis of the literature is needed to capture growth in recent research and address gaps in our understanding of factors that may optimize training parameters and treatment effects.

**Methods:**

Published randomized controlled trials comparing VR to conventional therapy were retrieved from seven electronic databases. Treatment effects (Hedge’s *g*) were estimated using a random effects model, with motor and functional outcomes between different protocols compared at the *Body Structure/Function*, *Activity*, and *Participation* levels of the International Classification of Functioning.

**Results:**

Thirty-three studies were identified, including 971 participants (492 VR participants). VR produced *small* to *medium* overall effects (*g* = 0.46; 95% CI: 0.33–0.59, *p* < 0.01), above and beyond conventional therapies. *Small* to *medium* effects were observed on *Body Structure/Function* (*g* = 0.41; 95% CI: 0.28–0.55; *p* < 0.01) and *Activity* outcomes (*g* = 0.47; 95% CI: 0.34–0.60, *p* < 0.01), while *Participation* outcomes failed to reach significance (*g* = 0.38; 95% CI: -0.29-1.04, *p* = 0.27). Superior benefits for *Body Structure/Function* (*g* = 0.56) and *Activity* outcomes (*g* = 0.62) were observed when examining outcomes only from purpose-designed VR systems. Preliminary results (*k* = 4) suggested *small* to *medium* effects for cognitive outcomes (*g* = 0.41; 95% CI: 0.28–0.55; *p* < 0.01). Moderator analysis found no advantage for higher doses of VR, massed practice training schedules, or greater time since injury.

**Conclusion:**

VR can effect significant gains on *Body Structure/Function* and *Activity* level outcomes, including improvements in cognitive function, for individuals who have sustained a stroke. The evidence supports the use of VR as an adjunct for stroke rehabilitation, with effectiveness evident for a variety of platforms, training parameters, and stages of recovery.

**Electronic supplementary material:**

The online version of this article (10.1186/s12984-018-0370-2) contains supplementary material, which is available to authorized users.

## Background

Stroke is one of the leading global causes of disability [1, 2], with over 17 million individuals worldwide sustaining a stroke each year [2]. Although stroke mortality is decreasing with improvements in medical technology [3], the neurological trauma resulting from stroke can be devastating, and the majority of stroke survivors have substantial motor [4, 5], cognitive [6–9] and functional rehabilitation needs [3, 10, 11], and much reduced quality of life [3, 12, 13]. Targeted rehabilitation can help address some of these post-stroke deficits, however, historically, many individuals, in particular patients with cognitive impairment, have difficulty engaging in standard therapies [14–16] at a level that will produce meaningful and lasting improvements [16–19]. Enriched and interactive rehabilitation programs are clearly needed to minimize functional disability [13, 20], increase participation in age-appropriate roles and activities [21], lead to greater motivation and treatment compliance [17, 22], and reduce the long-term expense of care in stroke survivors [20, 23, 24].

### Virtual reality

Virtual reality refers to simulated interactions with environments and events that are presented to the performer with the aid of technology. These so-called *virtual environments* may mirror aspects of the real world or represent spaces that are far removed from it, while allowing various forms of user interaction through movement and/or speech [25]. Virtual reality based rehabilitation, or *Virtual Rehabilitation* (VR), shows considerable promise as a safe, engaging, interactive, patient-centered and relatively inexpensive medium for rehabilitation training [26–31]. VR has the potential to target a wide range of motor, functional, and cognitive issues [23], affords methods that automatically record and track patient performance [32], and offers a high level of flexibility and control over therapeutic tasks [17, 18, 33]. This scalability allows patients to train at the highest intensity that would be possible for their individual ability [34], while keeping the experience of interaction with therapeutic tasks enjoyable and compelling [17, 29]. At the same time, VR may enable patients with a neurodisability (like stroke) to practice without excessive physical fatigue [32, 35] which otherwise may deter continued effort and engagement in therapy [36, 37].

Currently, there are two main types of VR: purpose-designed Virtual Environments (VE) and Commercial Gaming (CG) systems. Both types of systems can provide *augmented feedback*, additional forms of sensory feedback about the patient’s movement over and above the feedback that is provided as a natural consequence of the movement itself [11, 38]. VE systems are often designed by rehabilitation scientists (and others) to enhance the delivery of augmented feedback in order to develop the patient’s sense of position in space [39–41], to reinforce different movement parameters (like trajectory and endpoint) and reduce extraneous movements (e.g. excessive trunk displacement) [42, 43].

VE systems are also more likely to involve specially designed *tangible user interfaces* used in mixed reality rehabilitation systems [13] or training of daily functional activities [44]. By comparison, CG rehabilitation systems are typically “off-the-shelf” devices such as Wii (Nintendo), Xbox (Microsoft) and PlayStation (Sony), which have the advantage of being readily available and relatively inexpensive when compared with VE systems [11]. On the other hand, CG systems are typically designed for able-bodied participants and may not consider the physiological, motor, and cognitive aspects of recovery in rehabilitation, and may lack the scalability of purpose-designed VE systems [45].

### Systematic reviews comparing VE and CG systems

There is conflicting evidence about the relative effectiveness of VE- and CG-based VR systems. In a recent Cochrane review of VR following stroke [46], VE systems demonstrated a significant treatment effect on upper-limb function when compared to controls (*d* = 0.42; 95%CI: 0.07–0.76), while the effect for CG systems failed to reach significance (*d* = 0.50; 95%CI: -0.04-1.04); a caveat, however, was that only two of nine studies (22%) in these comparisons were CG-based. In contrast, a meta-analysis by Lohse and colleagues of VR following stroke [11] found no significant difference between VE (*g* = 0.43, based on 13 studies) and CG interventions (*g* = 0.76, based on three studies) on *Body Structure/Function* level outcomes. For *Activity* level outcomes, CG interventions showed a *large* but non-significant effect (*g* = 0.76, *p* = 0.14), but was based on only four of 26 studies (15%); VE interventions, however, showed a significant treatment effect (*g* = 0.54, *p* < .001). Taken together, these two reviews suggest benefits of VE systems, while previous analyses of CG treatment effects have been underpowered and inconclusive.

### Cognition and VR

Cognitive impairments, including difficulties in attention, language, visuospatial skills, memory, and executive function are common and persistent sequelae of stroke [14, 47] and exert considerable influence on rehabilitation outcomes [48]. Cognitive dysfunction may reduce the ability to (re-)acquire motor [25, 49–52] and functional skills [47], and decrease engagement and participation in rehabilitation program [48, 53]. While the important role of cognition in both conventional and VR-based rehabilitation is increasingly recognized [52–54] the impact of VR on cognitive function has not yet been formally evaluated in a quantitative review.

### Analysis of individual domains of functioning

The World Health Organization’s International Classification of Functioning, Disability, and Health (ICF-WHO [55]) is currently one of the most widely used classification systems. It is a foundation for understanding outcome effects in clinical practice [56] and the preferred means for translating clinical findings in a patient-centered manner [56]. Under the ICF-WHO, disability and functioning are seen to arise by the interaction of the health condition, the environment, and personal factors, and can be measured at three main levels: (i) *Body Structure/Function*, (ii) *Activity* (or skill), and (iii) *Participation*. The ICF-WHO has been used to classify outcome measures in studies of VR (for example [57]) and in recent systematic reviews [11, 58, 59]. A brief critique of these reviews reveals a number of important conclusions, but also some significant gaps in the research.

An early systematic review by Crosbie and colleagues [60] examined the efficacy of VR for stroke upon motor and cognitive outcomes. Of the 11 studies reviewed (up to 2005), only five addressed upper-limb function and two addressed cognitive outcomes. Overall, the review reported significant benefits of VR, but only three studies were RCTs and no effect size estimates were reported. At around the same time, a systematic review by Henderson and colleagues [61] showed that there was very good evidence that *immersive* VR was more beneficial than no therapy for upper-limb rehabilitation in adult stroke, but insufficient evidence for *non-immersive* VR. Comparisons with traditional physical therapy were less impressive, however.

A 2016 systematic review by Vinas-Diz and colleagues [62] included both controlled clinical trials and randomized controlled trials (RCTs) in stroke, and spanned 2009–2014. The review included 25 papers: four systematic reviews [19, 46, 63, 64] and 21 original trials. Evidence for treatment efficacy on upper-limb function was strong on a mix of measures like the Fugl-Meyer Test, Wolf Motor Function Test, and Motricity Index. However, a quantitative analysis of the effects was not undertaken, and important aspects of treatment implementation like dose and session scheduling were not formally examined.

A recent systematic review by Santos-Palma and colleagues [58] examined the efficacy of VR on motor outcomes for stroke using the ICF-WHO framework, covering work published up to June 2015. Of the studies deemed high quality, 20 examined outcomes at the *Body Structure/Function* level, 17 at the *Activity* level, and eight examined *Participation*. Intriguingly, positive outcomes were evident only at the *Body Structure/Function* level, while results for *Activity* and *Participation* were not conclusive. Unfortunately, only three studies addressed manual ability at the *Activity* level, which severely limited any evaluation of skill-specific effects.

In a combined systematic review and meta-analysis of 37 RCTs published between 2004 and 2013, Laver and colleagues [46] present a more comprehensive examination of the effects of VR on upper-limb function. As well, they classified outcomes broadly into upper-limb function, Activities of Daily Living (ADLs) and other aspects of motor function. In general, study quality was low, and the risk of bias high, in roughly one-half of the studies. Outcomes were significant for upper-limb function (*d* = 0.28) and ADLs (*d* = 0.43), but somewhat smaller than those reported by Lohse and colleagues [11]. Results for other aspects of motor function, including several at what may be considered the *Body Structure/Function* level, were non-significant. Dose varied considerably between studies, ranging from less than 5 h to more than 21 h in total. In general, studies that used higher doses (> 15 h of therapy) were reported as more effective. Unfortunately, results could not be pooled for cognitive outcomes, and the importance of additional treatment implementation parameters like training frequency and duration, and the impact of specific study design factors including the recovery stage of participants and type of control group (i.e. active vs passive) were not determined.

An updated systematic review by Laver and colleagues [65], included an additional 35 studies that reported outcomes for upper limb function and activity. A subset of only 22 studies that compared VR with conventional therapy showed no significant effect of VR on upper-limb function (*d* = 0.07). As well, there was no significant difference between higher (> 15 h of therapy), and lower levels of dose. However, when VR was used in addition to usual care (10 studies; 210 participants), there was a significant effect on upper-limb outcomes (*d* = 0.49). As before, no significant difference was shown between high and low dose studies. Unfortunately, analysis of cognitive outcomes, and moderator analyses including study quality, and implementation parameters (e.g., daily intensity, weekly intensity, treatment frequency, and total number of sessions) were not included in the updated review. As well, the assessment of study quality was limited to the 5-item GRADE system, the ICF classification system was not given full consideration, and no distinction was drawn between treatment as usual (TAU) and active control groups (TAU + some form of additional therapy).

Taken together, recent reviews on the use of VR for adult stroke show encouraging evidence of efficacy at the level of *Body Structure/Function*, but mixed results for *Activity* and ADLs, and a paucity of evidence bearing on *Participation*. The impact and effectiveness of VR on cognitive outcomes also remains poorly understood, despite the important role of cognitive dysfunction in learning and rehabilitation [17, 18], and increased evidence of interconnection between cognitive function and motor deficits at the *Body Structure/Function, Activity and Participation* levels of the ICF [52]. VE-based platforms have been suggested to be superior to CG approaches [46] in promoting motor function, but until recently there have been few CG studies available for analysis. As well, other design factors that may moderate treatment effects (like stage of recovery, control group type) have either not been explored or are too few in number to draw firm conclusions. There has been considerable variation in the total dose of VR therapy [46, 60], and no analysis has yet tested the dose-response relationship in moderator analyses. Finally, the bulk of conclusions have relied on qualitative synthesis, and there is a paucity of quantitative analysis of empirical data to inform opinion.

In view of limitations in past reviews and continued acceleration in VR the aim of our review was to conduct a systematic literature review and meta-analysis to re-evaluate the strength of evidence bearing on VR of upper-limb function and cognition in stroke. This review is critical given evidence that stroke rehabilitation needs to better optimize intervention techniques during the recovery windows that exist in the acute phase [66] and beyond. Focusing only on RCTs, we consider outcomes across levels of the ICF-WHO, and analyze the moderating effect of design factors and dose-related parameters.

## Methods

The current review was conducted and reported in accordance with the Preferred Reporting Items for Systematic Reviews and Meta-Analyses (PRISMA) statement [67], it should be noted that the protocol was not registered.

### Data sources and search strategy

Scopus, Cochrane Database, CINAHL, The Allied and Complementary Medicine Database, Web of Science, MEDLINE, Pre-Medline, PsycEXTRA, and PsycINFO databases were systematically searched from inception until 28 June 2017. Boolean search terms included the following: “*stroke*, *cerebrovascular disease*, or *cerebrovascular attack*” and “*Virtual reality*, Augment* *reality*, virtual gam*” (see Appendix for an example of the full MEDLINE search strategy).

### Inclusion and exclusion criteria

**Fig. 1:**
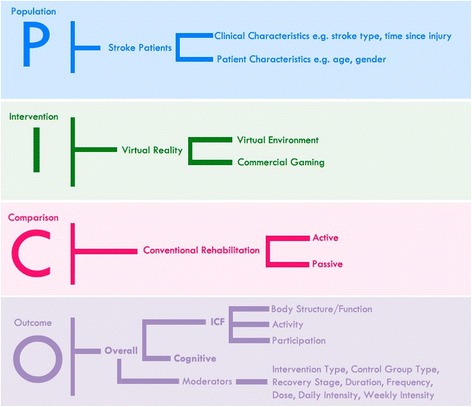
Population, Intervention, Comparison, Outcome (PICO) Question and the main variables included in the systematic literature review and meta-analysis

RCT studies published in English in peer-reviewed journals, utilizing a VR intervention to address either motor (upper-limb), cognitive, or activities of daily living in stroke patients were included in the current review (see Fig. 1). VR was defined as a type of user-computer interface that involves real-time simulation of an activity/environment, enabling the user to interact with the environment using motor actions and sensory systems. Comparison groups included “usual care”, “standard care” or “conventional therapy”, involving physical therapy and/or occupational therapy. Studies were excluded that applied a “hybrid” approach combining virtual reality with exogenous stimulation or robotics, targeted lower limb function, recruited a mixed study cohort including non-stroke participants, or did not utilize motor, cognitive, or participation outcome measures.

### Identification of relevant studies and data extraction

The eligibility assessment was performed independently using a standardized protocol by two of the authors (AA and JR). After deleting duplicate papers, the title and abstract of all articles were screened by the authors to assess suitability for inclusion. Those considered potentially eligible were read in full. In addition, reference lists of relevant reviews were searched by hand. The last hand search was performed 28 June 2017. For articles meeting inclusion criteria, data on study design, participant characteristics, and intervention outcomes were extracted by two of the authors (AA and JR). Disagreements between reviewers were resolved by consensus.

Extracted VR outcomes were organized according to the three levels of functioning classified by the ICF-WHO [55] including: (i) *Body Structure/Function*, which refers to physiological functions of body systems (e.g. Fugl Meyer Assessment); (ii) *Activity*, which refers to the execution of tasks or actions (e.g. Box and Blocks Test); and (iii), *Participation*, which refers to involvement in life situations (e.g. Motor Activity Log [57]).

### Quality assessment

Two authors (AA and PW) assessed the risk of bias of each included article using the Physiotherapy Evidence Database (PEDro) Scale [68]. The PEDro Scale rates methodological quality across 11 bias reducing items relating to the domains of Selection, Performance, Detection, Information, and Attribution biases [69]. Studies with PEDro total scores from 6 to 10 were considered *high* quality [70]; scores below 6 were considered *fair* quality. Disagreements between reviewers were resolved by consensus.

### Quantitative analysis

From the published manuscript, post-intervention means and standard deviations on each outcome measure, *p* values, and sample sizes for the experimental and control groups were entered into Comprehensive Meta-Analysis (CMA; Biostat, Englewood, NJ, USA) version 3.3.070. A random-effects model was used to compute the effect size estimate, Hedge’s *g*, a variation of Cohen’s *d* that corrects for small sample sizes. The magnitude of Hedge’s *g* was categorized as follows: *small* (≥0.2), *medium* (≥0.5) and *large* (≥0.8) [71]. Pooled effect sizes were calculated by aggregating the mean effect sizes weighted by each study’s sample size, and the 95% confidence intervals (CI) and *z* scores based on the overall mean and standard error. Meta-analysis was only performed in cases where there was more than one study in each group [72]. Effect size outcomes favoring VR were assigned a positive value while effects favoring the control condition (i.e. treatment-as-usual) had a negative value. Heterogeneity was formally assessed with the *I*^2^ statistic, where an *I*^2^ value greater than 50% indicated significant heterogeneity [71]. The risk of publication bias was assessed using the Classic fail-safe *N* and Egger’s regression test (2-tailed *p* value). Finally, moderator analyses were conducted using the *Q* statistic to estimate the likelihood of a given variable moderating observed effect sizes. A total of ten moderator variables were examined, including five design factors, and five implementation parameters (See Table 1).

**Table 1:** Moderators included in the analyses

Moderator Type	Definition
Design factors	
Intervention Type	Virtual Environment (VE) design or Computerized Gaming (CG) system
Simulation Type	Intervention targeting hand/finger function or interventions targeting overall upper-limb function
Study Quality	Moderate Quality (PEDro score ≤ 6) or High Quality (PEDro score > 6)
Recovery Stage	Sub-Acute (≤3 months post-stroke) or Chronic (> 3 months post-stroke)
Control Group Type	Passive (treatment as usual) or Active (treatment as usual + additional rehabilitation) comparison control group
Implementation Parameters	
Duration	Total number of sessions per intervention (low: < 15 sessions; high: ≥ 15 sessions)
Frequency	Number of sessions per week (low: ≤ 3 sessions; high: > 3 sessions)
Dose	Total number of minutes per intervention (low: < 400 min; medium: 400–800 min; high: > 800 min)
Daily Intensity	Minutes per session (low: ≤ 30 min; high: > 30 min)
Weekly Intensity	Total minutes of virtual rehabilitation per week (low: ≤ 100 min; high: > 100 min)

## Results

**Fig. 2:**
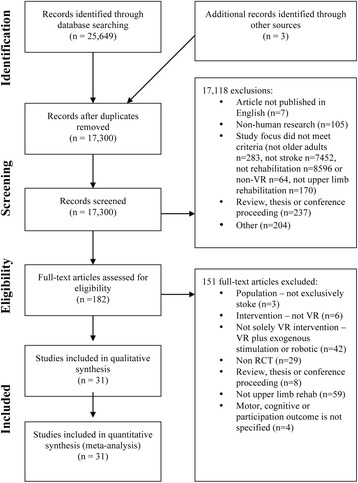
Four-phase Preferred Reporting Items for Systematic Reviews and Meta-Analyses (PRISMA) flow diagram, showing the process for identifying and screening of the articles for inclusion and exclusion in the systematic literature review and meta-analysis

Following removal of duplicates, 17,300 records were screened for eligibility. Following the selection process depicted in Fig. 2, a final sample of 31 articles was identified for inclusion in this review. Twenty-eight studies [13, 21, 44, 57, 73–96] utilized an upper-limb training intervention approach, one also aimed to improve cognitive function [97], and two studies [53, 54] targeted cognitive function alone. The pool of studies included work conducted in the UK, Korea, Spain, USA, Brazil, Israel, Sweden, Australia, and New Zealand (see Table 2). Of the 31 articles, two presented two separate studies for analysis [21, 91], providing a total of 33 independent studies. All studies used an RCT design, comparing 492 participants receiving VR (per study *M* = 14.9, *SD* = 10.9) with 479 participants receiving Conventional Therapy (CT; per study *M* = 14.5, *SD* = 11.4).

**Table 2:** Characteristics of the included studies

	Participant Characteristics						Study Design			
First Author, Year (Reference)	N (VR/CT)	Mean Age at Intervention	Time Since Stroke (Weeks)	Recovery stage	Stroke Type	Cohort Origin	VR Type	Implementation Parameters	Control Type	Outcomes
Assis, 2014	4/4	55.0	269.6	Chronic	NR	Brazil	VE	One hour training once a week, for 4 weeks	Active	Body Function
Broeren, 2008^a^	11/11	68.0	306.1	Chronic	Mixed	Sweden	VE	Forty-five minutes training, 3 times a week for 4 weeks	Passive	Activity
Chen, 2015 *Study 1*	8/8	49.2	36.3	Chronic	NR	Taiwan	CG	Thirty minutes training 3 times a week for 8 weeks	Active	Body Function, Activity
Chen, 2015 *Study 2*	8/8	53.3	37.8	Chronic	NR	Taiwan	CG	Thirty minutes training 3 times a week for 8 weeks	Active	Body Function, Activity
Choi, 2014^c^	10/10	64.7	NR	NR	Mixed	Korea	CG	Thirty minutes training, 5 times a week for 4 weeks	Active	Body Function, Activity, Cognitive
Crosbie, 2012^a,c^	9/9	60.4	46.9	Chronic	NR	UK	VE	Thirty minutes training, 3 times a week for 3 weeks	Active	Activity
da Silva Cameirão, 2011^a,b^	8/8	61.4	1.9	Subacute	Mixed	Spain	VE	*Study 1:* Twenty minutes training, 3 times a week for 5 weeks.*Study 2:* Twenty minutes training, 3 times a week for 12 weeks.	Active	Body Function, Activity
da Silva Ribeiro, 2015	15/15	53.3	222.7	Chronic	NR	Brazil	CG	One hour training twice a week, for 8 weeks	Active	Body Function, Activity
Duff, 2010	11/10	68.5	56.3	Chronic	Mixed	USA	VE	One hour training 3 times a week, for 4 weeks	Active	Body Function, Activity, Participation
Gamito, 2015	10/10	55.0	NR	NR	NR	Portugal	VE	One hour training 3 times a week, for 6 weeks	Passive	Cognitive
Givon 2015	19/18	59.4	146.0	Chronic	Mixed	Israel	CG	One hour training 2 times a week, for 12 weeks	Active	Body Function, Activity
Housman, 2009^b^	15/16	55.3	427.8	Chronic	Mixed	USA	VE	One hour training 3 times a week, for 8 weeks	Passive	Body Function, Participation
In, 2012^a^	11/8	64.0	57.9	Chronic	Mixed	Korea	VE	Thirty minutes training, 5 times a week for 4 weeks	Active	Body Function, Activity
Kihoon, 2012^a^	15/14	63.9	NR	NR	NR	Korea	CG	One hour training 5 times a week, for 4 weeks	Passive	Body Function, Cognitive
Kim, 2011^b^	15/13	64.2	3.0	Subacute	Mixed	Korea	CG	Thirty minutes training, 3 times a week for 4 weeks	Passive	Activity, Cognitive
Kim, 2012^a,b^	10/7	48.2	54.8	Chronic	Mixed	Korea	CG	Thirty minutes training, 3 times a week for 3 weeks	Passive	Activity
Kiper, 2011^a,b^	40/40	64.0	25.0	Chronic	Mixed	Italy	VE	One hour training 5 times a week, for 4 weeks	Active	Body Function, Activity
Kiper, 2014	23/21	64.3	18.3	Chronic	Mixed	Italy	VE	One hour training 5 times a week, for 4 weeks	Active	Body Function, Activity
Kong, 2016	31/33	57.5	1.96	Subacute	Mixed	Singapore	CG	One hour training 4 times a week, for 3 weeks	Passive	Body Function, Activity
Kottink, 2014	8/10	61.4	173.1	Chronic	Mixed	The Netherlands	VE	Thirty minutes training, 3 times a week for 6 weeks	Active	Body Function, Activity
Kwon, 2012 ^a,b^	13/13	57.5	34.9	Chronic	Mixed	Korea	CG	Thirty minutes training, 5 times a week for 4 weeks	Passive	Body Function, Activity
Lee, 2013	7/7	74.1	33.8	Chronic	Mixed	Korea	CG	Thirty minutes training, 3 times a week for 6 weeks	Passive	Body Function, Activity
Levin, 2012	6/6	59.0	165.3	Chronic	Mixed	Israel	VE	Forty-five minutes training, 3 times a week for 3 weeks	Active	Body Function, Participation
Piron, 2009 ^a,b,c^	18/18	65.2	57.9	Chronic	Ischemic, MCA	Italy	VE	One hour training 5 times a week, for 4 weeks	Active	Body Function
Piron, 2010 ^a,b,c^	27/23	60.5	66.2	Chronic	Ischemic, MCA	Italy	VE	One hour training 5 times a week, for 4 weeks	Active	Body Function, Activity
Saposnik, 2016	59/62	62.0	3.7	Subacute	Ischemic	Canada, Argentina, Peru, Thailand	CG	One hour training 5 times a week, for 2 weeks	Passive	Body Function, Activity
Shin, 2014	9/7	49.3	10.3	Subacute	NR	Korea	VE	Twenty minutes training, 5 times a week for 2 weeks	Active	Body Function, Activity
Shin, 2015 ^c^	16/16	54.0	26.2	Chronic	NR	Korea	VE	Thirty minutes training, 5 times a week for 4 weeks	Active	Body Function, Activity
Sin, 2013^b^	18/17	73.7	34.1	Chronic	Mixed	Korea	CG	Thirty minutes training, 3 times a week for 6 weeks	Passive	Body Function, Activity
Standen, 2017	9/9	61.0	NR	NR	NR	UK	VE	Twenty minutes training, 3 times a day for 8 weeks	Passive	Body Function, Activity, Participation
Yavuzer, 2008 ^a,b,c^	10/10	61.1	17.2	Chronic	NR	Turkey	CG	Thirty minutes training, 5 times a week for 4 weeks	Passive	Activity
Yin, 2014	11/10	58.4	2.3	Subacute	NR	Singapore	VE	Thirty minutes training, 5 times a week for 2 weeks	Passive	Body Function, Activity, Participation

Note: *CG* Commercial Gaming, *NR* Not Reported, *VE* Virtual Environment

Articles included in previous reviews: ^a^Article included in the Lohse review [11] ^b^Article included in the Laver review [46]; ^c^Article included in the Palma review [58]

### Participant characteristics

Sample sizes ranged from 4 to 62 participants per group. Eight studies had less than 10 participants in the VR group [21, 57, 76, 79, 81, 83, 84, 90], while only five studies had over 20 participants (range, 20–59) in the VR group [74, 77–79, 86] (see Table 2). The average age was 60.0 years (*SD* = 6.3 years, range 48.2–74.1). The average time post-stroke for each study (based on 29 independent studies, four studies [54, 90, 91, 97] did not report time post-stroke) varied considerably from 1.9 weeks to 427.8 weeks (*M* = 79.6 weeks, *SD* = 105.2). This included seven studies [21, 44, 53, 74, 76, 86, 91] (21%) conducted during the sub-acute (≤ three months) stage (range 1.9–10.3 weeks, *M* = 3.86 weeks, *SD* = 3.23), while the remainder completed VR interventions during the chronic (> 3 months post-stroke) stage (range 17.2–427.8 weeks, *M* = 127.40 weeks, *SD* = 132.5). Seventeen studies [13, 21, 53, 57, 74, 77, 79–82, 85, 87, 91, 92, 94] included both ischemic and hemorrhagic stroke patients, three included only ischemic stroke patients [78, 86, 93], and 11 did not report specific details about stroke type. Only three studies [21, 74] reported data on stroke severity, two utilizing the National Institutes of Health Stroke Scale (NIHSS) and one study [86] used the Canadian Neurological Scale of Stroke Severity.

### VR and control interventions

Of the 33 independent studies, 19 used a VE approach and 14 studies evaluated a CG-based therapy (see Table 3). VE interventions involved either video capture or tabletop systems. The former required the patient to be seated in front of a wall display while grasping a sensor, such as the Reinforced Feedback in Virtual Environment system [77–79] and the Rehab Master game-based VE system [75, 76]. Tabletop systems involved multitouch display technologies (e.g. [92, 94]), requiring finger touch response [94] or the manipulation of tangible user interfaces. CG therapies included Wii (Nintendo [73, 74, 80, 83, 86, 91]), Xavix [83], EyeToy (PlayStation [88]), IREX system [53, 82, 97], Xbox Kinect [81, 89], or a combination of systems [95]. All but two intervention programs (93%) took place in a hospital, one [90] was home-based and another provided rehabilitation at a local community center [92]. Only one study [85] reported on the number of repetitions per session.

**Table 3:** Description of the virtual rehabilitation interventions, conventional control group therapies, and additional control treatments, when applicable

First Author, Year	Virtual Rehabilitation Intervention	Control Group Intervention
Assis, 2014	VE: NeuroR augmented reality system, with *virtual shoulder* exercises to extend the upper-limb + TAU	TAU: Standard Therapy (NR) Ancillary: Relaxation session instructed by physiotherapist followed by shoulder movement using both unaffected and injured arms assisted by the physiotherapist and also without the physiotherapist assistance.
Broeren, 2008	VE: Semi-immersive table-top workbench with haptic device and 3D computer games + TAU	TAU: Usual activities at the centre, which included different social activities, creative crafts and physical activities. Ancillary: NA
Chen, 2015	Study 1 – VE: Wii Nintendo games bowling and boxing games + TAUStudy 2 – VE: XaviX games bowling and ladder climbing games + TAU	TAU: At least one hour of physio- and occupational therapy per week. Ancillary: Two traditional devices, the Curamotion exerciser and the climbing board and bar
Choi, 2014	CG: Wii Nintendo games: swordplay, table tennis, and canoe + TAU	TAU: Standard Therapy (NR) Ancillary: OT that involved goal-oriented and highly repetitive trainings for that involved composed of stretching and strengthening exercises using full range of motion of the upper extremity, which was a task-oriented therapy for the ADL, fine motor training, and sensory motor recover
Crosbie, 2012	VE: 3D environment system, that included a desktop computer, a head-mounted display unit, a motion tracking system and sensors with tasks focused on reaching and grasping. + TAU	TAU: Standard physiotherapy Ancillary: Delivered by a physiotherapist, experienced in stroke rehabilitation, and followed a programme of techniques, which included muscle facilitation, stretching exercises, strengthening activities and the inclusion of the more affected upper limb in functional tasks
da Silva Cameirão, 2011	Study 1 - VE: Rehabilitation Gaming System that captures upper limb movements through color detection; two data gloves to capture finger flexure creating a virtual environment where an avatar mimics the movements of the user. Tasks aimed to target speed, range of motion, grasp and release + TAU for 5 weeks. Study 2 - VE: Rehabilitation Gaming System that captures upper limb movements through color detection; two data gloves to capture finger flexure creating a virtual environment where an avatar mimics the movements of the user. Tasks aimed to target speed, range of motion, grasp and release + TAU for 12 weeks.	TAU: Two weekly physiotherapy sessions Ancillary: involved release or non-specific interactive Wii Nintendo games either intense occupational therapy targeting object displacement, grasp and.
da Silva Ribeiro, 2015	CG: Wii Nintendo games: tennis, hula-hoop, soccer and boxing. + TAU	TAU: NR Ancillary: Conventional physiotherapy that included 10-mintes of upper and lower limb stretching or muscles and trunk, passive, active-assisted, and active-resisted mobilisation of the trunk (10 min), straightening and balance reactions with rapid shifts (10 min), scapular mobilisation (5 min), active or active-assisted upper-limb diagonal movements (15 min) and grasping activities (10 min)
Duff, 2010	VE: Adaptive mixed reality rehabilitation system + TAU	TAU: Standard Therapy (NR) Ancillary: UL therapy: pegboard reaching tasks, bead threading reaching tasks, cone reaching tasks, and ROM and coordination exercises
Gamito, 2015	VE: Cognitive stimulation with Serious Games mobile technology, that included several daily live activities e.g. buying items, findings way to the minimarket, finding a virtual character. + TAU	TAU: Standard Therapy (NR) Ancillary: NA
Givon 2015	CG: Microsoft Xbox Kinect, Sony PlayStation 2 Eyetoy, Sony PlayStation 3 MOVE, Nintendo Wii Fit and the SeeMe VR systems. + TAU	TAU: Standard Therapy (NR) Ancillary: Participants were then divided into pairs or triads to perform functional activities such as picking up and transferring objects from one side of the room to the other.
Gyuchang, 2013	CG: Xbox Kinect + TAU	TAU: Standard occupational therapy Ancillary: NA
Housman, 2009	Therapy Wilmington Robotic Exoskeleton (T-WREX) a passive (non-robotic) arm that provides support for the arm against gravity and measures arm movement and traces hand grasp as users interact with computer games	TAU: Standard Therapy (NR) Ancillary: Conventional semiautonomous training
In, 2012	VE: Virtual Reality Reflection Therapy program + TAU	TAU: Standard Therapy (NR)Ancillary: The control group received the same treatment as intervention group, but the monitor was off.
Kihoon, 2012	CG: Interactive Rehabilitation and Exercise System (IREX) + TAU	TAU: Standard Therapy (NR) Ancillary: NA
Kim, 2011	CG: Interactive Rehabilitation and Exercise System (IREX) + TAU	TAU: Computer assisted cognitive rehabilitation Ancillary: NA
Kim, 2012	CG: Nintendo Wii Tennis and boxing games	TAU: Previous therapy (not specified), no therapy at the time of intervention Ancillary: NA
Kiper, 2011	VE: Reinforced feedback in virtual environment (RFVE) system + TAU	TAU: Standard Therapy (NR) Ancillary: Additional Standard Therapy (tailored to individual needs)
Kiper, 2014	VE: Reinforced feedback in virtual environment (RFVE) system + TAU	TAU: Standard Therapy (NR) Ancillary: Additional Standard Therapy (tailored to individual needs)
Kong, 2016	CG: Nintendo Wii Tennis, golf, baseball, table tennis, basketball, cycling, sword play, airplane flight control and boxing games (Wii Sports and Wii Sports resort packages) + TAU	TAU: Standard Therapy consisted of physical and occupational therapy one hour a day, Monday to Friday. Ancillary: NA
Kottink, 2014	VE: The Furball Hunt table-top rehabilitation game + TAU	Standard therapy tailored to individual needs Ancillary: Exercises required reaching for targets, such as objects positioned on a table top, or using specific, non-(electro) mechanical equipment (bow, pegs in holes, placing disks, etc.)
Kwon, 2012	CG: Interactive Rehabilitation and Exercise System (IREX) + TAU	TAU: Standard physical and occupational therapy Ancillary: NA
Lee, 2013	CG: Xbox Kinect + TAU	TAU: Standard occupational therapy Ancillary: NA
Levin, 2012	VE: Virtual games and a virtual supermarket (e.g., Birds & Balls, Soccer, Volleyball, VMall) + TAU	TAU: NR Ancillary: Occupational therapy, including exercises involving reaching for and holding cones, cups, and other objects in all motion planes with and without external loading
Piron, 2009	VE: Therapist telerehabilitation equipment (VRRS.net®), participant can observe his/her movement on the screen (augmented feedback), and observe the correct trajectory pre-recorded in the virtual scene (virtual teacher) + TAU	TAU: NR Ancillary: Conventional physical therapy, with patients performing specific exercises (e.g. touching different targets arranged in a horizontal plane in front of them) with a strategy of progressive complexity.
Piron, 2010	VE: Reinforced feedback in a virtual environment (RFVE) system + TAU	TAU: Conventional physical therapy Ancillary: The CT program was based on Bobath principles. The patients performed specific exercises with the upper limb with progressive complexity. First, the patients were asked to control isolated motions without postural control; subsequently, postural control was included; and finally, complex motion with postural control was practiced.
Saposnik, 2016	CG: Nintendo Wii, Wii sports and Game Party 3 packages + TAU	TAU: Standard Therapy (NR) Ancillary: Recreational computer-generated activities (passive control)
Shin, 2014	VE: The RehabMaster game-based system + TAU	TAU: Standard Therapy (NR) Ancillary: Occupational therapy
Shin, 2015	VE: The RehabMaster game-based system + TAU	TAU: Standard Therapy (NR) Ancillary: Occupational therapy
Sin, 2013	CG: Xbox Kinect Microsoft + TAU	TAU: Standard Therapy (NR) Ancillary: NA
Standen, 2017	VE: Home-based VR system with three games (Spacerace, Spongeball, Balloonpop)	TAU: Previous therapy, no therapy at the time of intervention Ancillary: NA
Yavuzer, 2008	CG: Playstation EyeToy games (Kung-foo, goal attack, MrChef, Dig and HomeRun) + TAU	TAU: Physiotherapy, occupational therapy, speech therapy (if needed) Ancillary: “Sham therapy” (not specified, passive control)
Yin, 2014	VE: VE of a local supermarket setting + TAU	TAU: Standard Therapy (NR) Ancillary: NA

Note: *CG* Commercial Gaming, *NA* Not Applicable, *NR* Not Reported, *TAU* Treatment as Usual, *VE* Virtual Environment

All VR and CT group participants received CT. In most of the included studies, this “treatment as usual” was only described in limited terms, but typically involved aspects of either physio- or occupational therapy (see Table 3). In 21 studies, CT group participants also received additional rehabilitation interventions, to match the additional time in therapy provided to participants randomized to VR. These so-called “active” control group interventions included, for example, additional physio- and occupational therapy [83], or additional standard therapy tailored to individual needs [77, 79, 93] (see Table 3). In contrast, 12 studies utilized “passive” control groups that received no additional intervention beyond treatment as usual.

### Dose and session scheduling

For all VR approaches combined, the mean overall *Dose* was 685 min (*SD* = 355, range 200–1440 min), with a mean *Daily Intensity* of 42 min (*SD* = 15, median 30, range 20–60 min) and *Weekly Intensity* of 153.9 min (*SD* = 80.38, median = 135, range 60–800). The mean *Frequency* was three sessions a week (range one-five sessions), and the median *Duration* was 18 sessions (range, 4–36 sessions).

### ICF-WHO outcomes

Twenty-seven studies reported *Body Structure/Function* level outcomes, with the Fugl-Meyer Assessment-Upper Extremity (FMA-UE) as the most common instrument (21 studies). An additional study [95] utilized the FMA-UE to classify baseline participant characteristics but did not include it as an outcome measure. Twenty-nine studies reported *Activity* level outcomes, most commonly using the Box and Blocks Test (seven studies), Functional Independent Measure (eight studies), and Barthel Index (six studies). *Participation* level outcomes were reported by five studies, most often utilizing the Motor Activity Log instrument (four studies). Only four studies [53, 54, 91, 97] reported data on cognitive outcomes (see Table 4), each of these studies reported data on multiple cognitive outcomes, and all of these were included in the analyses (Table 5).

**Table 4:** Outcome measures included in the data analysis

	Body Structure and Function			Activity					Participation	Cognitive
First Author, Year	FMA-UE	MAS	Other outcomes	BBT	ARAT	FIM	BI	Other outcomes		
Assis, 2014	✓									
Broeren, 2008				✓						
Chen, 2015	✓			✓		✓				
Choi, 2014	✓		MFT; Grip Strength	✓			✓			A-CPT Correct Detection, Reaction Time, Commission Error; V-CPT Correct Detection, Reaction Time
Crosbie, 2012					✓			MI		
da Silva Cameirão, 2011	✓		FMA arm; FMA hand/wrist				✓	CAHAI; MI		
da Silva Ribeiro, 2015	✓		FMA Overall, Physical Functioning (overall motor functioning					SF-36 Social Aspects, Vitality		
Duff, 2010	✓		FMA range of motion, pain, sensation					SIS; WMFT total; WMFT time.	MAL Amount of Use, Quality of Movement	
Gamito, 2015										TP; WMS III
Givon 2015			Grip Strength Weaker Hand; Gait Speed (Motor Overall)		✓					
Housman, 2009	✓		Grip strength					Racho level (UL); Racho Speed (UL)	MAL Amount of Use, Quality of Movement	
In, 2012	✓	✓	MFT	✓				JHFT		
Kihoon, 2012								WMFT total, hand, arm		MVPT Reaction time in seconds; MVPT score; Visual discrimination; Visual memory; visual closure
Kim, 2011							✓	MI		VCPT; ACPT; WCW; CCW; FDST; BDST; FVST; BVST; ViLT; VeLT; TMT-A; TOL
Kim, 2012		✓	PASS Motor overall			✓				
Kiper, 2011	✓	✓				✓				
Kiper, 2014	✓		Mean Duration of Movement – time; peak; speed			✓				
Kong, 2016	✓				✓	✓				
Kottink, 2014	✓				✓					
Kwon, 2012	✓		MFT; FMA arm, hand, wrist				✓	K-MBI Self Care		
Lee, 2013	✓		MMT Muscle Strength;			✓				
Levin, 2012	✓		RPSS	✓				WMFT	MAL Amount of Use, Quality of Movement	
Piron, 2009	✓	✓								
Piron, 2010	✓					✓				
Saposnik, 2016				✓		✓	✓	WMFT		
Shin, 2014	✓						✓			
Shin, 2015	✓							SF-36 Role limitation due to physical problems; SF-36 Vitality		
Sin, 2013	✓			✓						
Standen, 2017			NHPT					NEADL; WMFT	MAL Amount of Use, Quality of Movement; MAL-activities	
Yavuzer, 2008								Brunnstorm stages hand, Upper Extremity; FIM Self Care		
Yin, 2014	✓	✓			✓				MAL Amount of Use, Quality of Movement	

Note: *ACPT* Auditory continuous performance test, *AF* Augmented Feedback, *ARAT* Action Research Arm Test, *AS* Ashworth scale, *BDST* Backward digit span test, *BVST* Backward visual span test, *BI* Barthel Index, *BBT* Box and Blocks test, *CCW* Color of word in word-color test, *CAHAI* Chedoke Arm and Hand Activity Inventory, *CSI* Composite Spasticity Index, *FMA* Fugl-Meyer Assessment, *FMA-UE* Fugl-Meyer Assessment-Upper Extremity FDST: Forward digit span test, *FVST* Forward visual span test, *JHFT* Jebsen Hand Function Test, *K-MBI* Korean version of the Modified Barthel Index, MAS: Modified Ashworth Scale, *MAL* Motor Activity Log, *MFT* Manual Function Test, *MI* Motricity Index, *TPT* Toulouse–Pieron Test, *VCPT* Visual continuous performance test, *ViLT* Visual learning test, *VeLT* Verbal learning test, *TMT-A* Trail Making Test-A, *TOL* Tower of London test, *QOM* Quality of Movement, *RPSS* Reaching Performance Scale for Stroke, *WMFT* Wolf Motor Function Test, *WMS-III* Wechsler Memory Scale Third Edition, *WCW* Word in word-color test

**Table 5:** PEDro Scale risk of bias ratings for the included studies

First Author, Year	C1	C2	C3	C4	C5	C6	C7	C8	C9	C10	C11	TOTAL
Assis, 2014	1	1	0	0	0	0	0	1	1	1	1	6
Broeren, 2008	1	1	0	1	0	0	0	1	0	0	1	5
Chen, 2015	1	0	0	1	0	0	1	1	1	1	1	7
Choi, 2014	1	1	1	1	0	0	1	1	1	1	1	9
Crosbie, 2012	1	1	1	1	0	0	1	1	1	1	1	9
da Silva Cameirão, 2011	1	1	0	0	0	0	1	0	0	1	1	5
da Silva Ribeiro, 2015	1	1	1	1	0	0	1	1	0	1	1	8
Duff, 2010	1	1	0	1	0	0	1	0	0	1	1	6
Gamito, 2015	1	1	0	0	0	0	0	1	1	1	1	6
Givon, 2015	1	1	0	1	0	0	1	1	1	1	1	8
Housman, 2009	1	1	1	1	0	0	1	1	0	1	1	8
In, 2012	1	1	0	1	0	0	0	0	0	1	1	5
Kihoon, 2012	1	1	0	1	0	0	0	1	0	1	1	6
Kim, 2011	1	1	0	1	0	0	0	1	1	1	1	7
Kim, 2012	1	1	0	1	0	0	0	0	0	1	1	5
Kiper, 2011	1	1	0	1	0	0	0	1	1	1	1	7
Kiper, 2014	1	1	0	1	0	0	0	1	1	1	1	7
Kong, 2016	1	1	1	1	0	0	1	1	1	1	1	9
Kottink, 2014	1	1	0	1	0	0	1	1	0	1	1	7
Kwon, 2012	1	1	0	1	1	0	1	1	1	1	1	9
Lee, 2013	1	1	0	0	0	0	0	1	1	1	1	6
Levin, 2012	1	1	0	1	0	0	1	1	0	1	1	7
Piron, 2009	1	1	1	0	0	0	1	1	1	1	1	8
Piron, 2010	1	1	1	0	0	0	1	1	1	1	1	8
Saposnik, 2016	1	1	0	1	0	0	1	1	1	1	1	8
Shin, 2014	1	1	0	1	0	0	1	1	0	1	1	7
Shin, 2015	1	1	0	1	0	0	1	1	0	1	1	7
Sin, 2013	1	1	0	1	0	0	1	1	0	1	1	7
Standen, 2017	1	1	1	1	0	0	0	1	0	1	1	7
Yavuzer, 2008	1	1	1	1	0	0	1	1	1	1	1	9
Yin 2014	1	1	1	1	0	0	0	0	0	1	1	6

Note: “1” indicates a study met the criteria, “0” indicates there was not enough information to make an assessment or the criterion was not met. C1 = Eligibility criteria were specified. C2 = Subjects were randomly allocated to groups (in a crossover study, subjects were randomly allocated an order in which treatments were received). C3 = Allocation was concealed. C4 = The groups were similar at baseline regarding the most important prognostic indicators. C5 = There was blinding of all subjects. C6 = There was blinding of all therapists who administered the study. C7 = There was blinding of all assessors who measured at least one key outcome. C8 = Measures of at least one key outcome were obtained from more than 85% of the subjects initially allocated to groups. C9 = All subjects for whom outcome measures were available received the treatment or control condition as allocated or, where this was not the case, data for at least one key outcome was analyzed by “intention to treat.” C10 = The results of between-group statistical comparisons are reported for at least one key outcome. C11 = The study provides both point measures and measures of variability for at least one key outcome

### Risk of bias

The methodological quality of included studies was generally *high* (see Table 4), with an average PEDro total score of 7.06 (*SD* = 1.26, range 5–9). Eligibility criteria were specified in all studies, and all but one study [83] specified random allocation of participants. However, despite more rigorously focusing only on RCT designs, However, despite more rigorously focusing only on RCT designs, four [21, 80, 85, 92] of the included studies were rated only *fair* quality, due to the omission of concealed allocation, blinding, and intention to treat analyses. In addition, the Egger’s intercept value for all outcomes combined was 1.23, *p* = 0.02 (two-tailed), suggesting pronounced asymmetry and an increased likelihood that smaller studied tended to report larger than average effects [98]. To minimize the risk of publication bias all reported effect size outcomes were based on a random-effects model to give more weight to larger trials [99].

### Main effects of VR after stroke

**Fig. 3:**
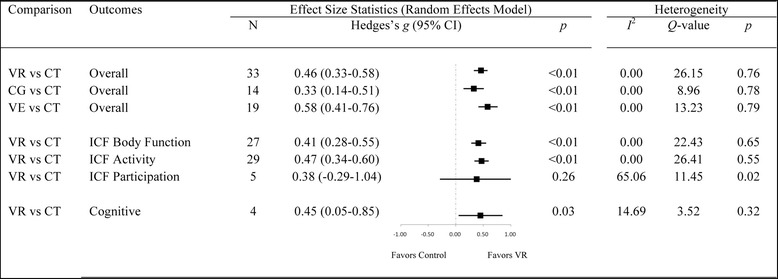
Forest plot showing the main effect-sizes of Virtual Rehabilitation after stroke on the motor, functional, and cognitive outcomes combined; the three levels of the International Classification of Functioning (Body Function outcomes included Fugl-Meyer Assessment-Upper Extremity and Modified Ashworth Scale; Activity outcomes included Box and Blocks Test; Participation outcomes included Motor Activity Log and Quality of Movement); and cognitive outcomes using the random-effects model. Notes: CG: Computerized Gaming; CI: Confidence Intervals; CT: Conventional Treatment; ICF: International Classifacation of Functioning; VE: Virtual Environment; VR: Virtual Rehabilitation

For all outcomes combined (see Fig. 3 and Additional file 1: Figure S1), the average effect size for VR interventions was *small* to *medium* (*g* = 0.46; 95% CI: 0.33–0.59, *p* < 0.01), with significant benefit of VR compared to CT. The overall fail-safe *N* was high at 439, and heterogeneity minimal (*I*^2^ = 0%), suggesting a robust finding. Both VE and CG approaches were significantly more effective than CT, with an average *small* effect size for CG (*g* = 0.33; 95% CI: 0.14–0.51, *p* < 0.01), and an average *medium* effect size for VE interventions (*g* = 0.58; 95% CI: 0.41–0.76, *p* < 0.01). Moderator analysis confirmed the difference between VE and CG-based approaches was statistically significant [*Q*(1) = 3.96, *p* = 0.047].

The average effect size for cognitive outcomes was *small* but significant (*g* = 0.45, 95% CI: 0.02–0.88, *p* = 0.04). Heterogeneity between studies was minimal (*I*^2^ = 14.69%), but the fail-safe *N* was only 2, suggesting a tenuous finding. For upper-limb motor and functional outcomes, data was examined at each of the three ICF-WHO levels (see Fig. 3 and Additional file 2: Figure S2). *Small* overall to *medium* effects were observed on *Body Structures/Function* (*g* = 0.41; 95% CI: 0.28–0.55; *p* < 0.01) and *Activity* outcomes (*g* = 0.47; 95% CI: 0.34–0.60, *p* < 0.01), while *Participation* outcomes were non significant (*g* = 0.38; 95% CI: -0.29-1.04, *p* = 0.27).

### Moderator analysis

**Fig. 4:**
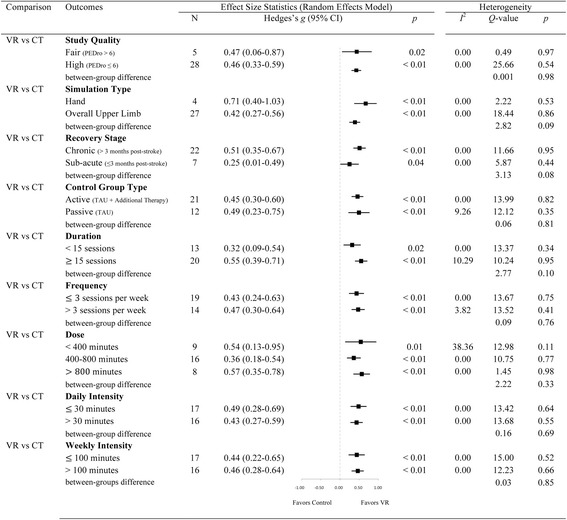
Forest plot showing the main moderator analyses of Virtual Rehabilitation outcomes after stroke using the random-effects model. Note: AR: Additional Rehabilitation; CI: Confidence Intervals; CT: Conventional Treatment; TAU; Treatment As Usual; VR: Virtual Rehabilitation

Moderator analysis (see Fig. 4) found no significant difference in the overall outcomes of interventions that utilized an active or passive control group [*Q*(1) = 0.05, *p* = 0.83], and between moderate and high quality studies [*Q*(1) = 0.001, *p* = 0.98], and between studies with low and high sample size [*Q*(1) = 0.67, *p* = 0.41]. Moreover, there was no significant difference in overall outcomes for patients receiving VR during either the sub-acute or chronic stage [*Q*(1) = 2.39, *p* = 0.12], and between interventions that focused specifically on hand function or overall upper-limb function [*Q*(1) = 2.82, *p* = 0.09].

Different levels of dose (high, medium, low) had no significant effect on the overall effect [*Q*(2) = 2.22, *p* = 0.33]. Variations in daily intensity [*Q*(1) = 0.16, *p* = 0.70], frequency [*Q*(1) = 0.67, *p* = 0.71], weekly intensity [*Q*(1) = 0.03, *p* = 0.85] and duration [*Q*(1) = 2.77, *p* = 0.10] also had no significant impact.

Meaningful comparisons could not be performed between different levels of severity (determined using gold standard FMA-UE outcome measure); there was only a single study that used a group of mild severity [82]. The larger (moderate-severe) group clustered tightly around a mean severity of 34.9 (*SD*: 8.9). When the mild severity study [82] was pulled-out from the overall analysis, the overall effect for all outcomes combined remained *small* (*g* = 0.47; 95% CI: 0.34–0.60, *p* < 0.01), with significant benefit of VR compared with CT.

**Fig. 5:**
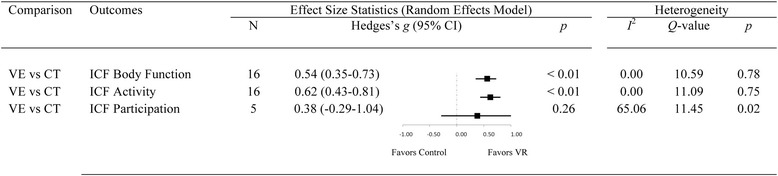
Forest plot showing the main effect-sizes of Virtual Environment therapy after stroke on the three levels of the International Classification of Functioning using the random-effects model. Body Function outcomes included Fugl-Meyer Assessment-Upper Extremity and Modified Ashworth Scale; Activity outcomes included Box and Blocks Test; Participation outcomes included Motor Activity Log and Quality of Movement. Note: CI; Confidence Intervals; CT: Conventional Treatment; ICF: International Classifacation of Functioning; VE: Virtual Environment

On the basis of the statistically significant advantage for VE approaches relative to CG designs, treatment effects for VE-based rehabilitation alone were also analyzed at each ICF-WHO level (see Fig. 5). There was a *medium* effect overall on *Body Structure/Function* (*g* = 0.54; 95% CI: 0.35–0.73; *p* < 0.01), and a *medium* to *large* effect on *Activity* (*g* = 0.62; 95% CI: 0.43–0.81, *p* < 0.01). The overall effect on *Participation* was unchanged as no CG approaches examined outcomes in this ICF-WHO domain. Within-group heterogeneity was minimal for *Activity* (*I*^2^ = 0%) and *Body Function* (*I*^2^ = 0%) outcomes, and *large* for *Participation* outcomes (*I*^2^ = 65%).

### Follow-up data

**Fig. 6:**
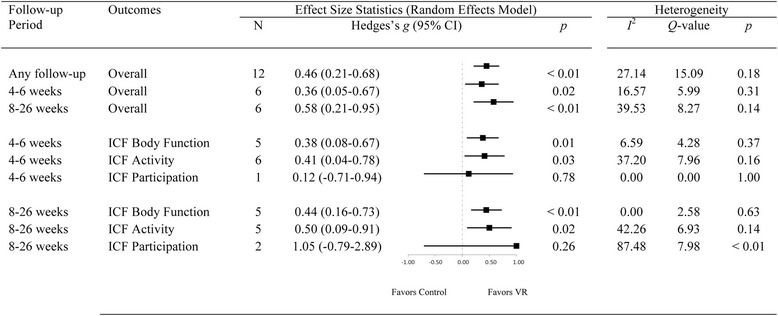
Forest plot showing the follow-up effects of Virtual Rehabilitation after stroke on the motor, functional, and cognitive outcomes combined using the random-effects model Note: CI; Confidence Intervals; ICF: International Classifacation of Functioning

Twelve studies also included follow-up data: six studies re-assessed outcomes four to six weeks after intervention [44, 76, 79, 86, 94, 96] and six studies re-assessed outcomes eight to 26 weeks later [74, 87, 88, 90, 93, 95]. Both CG [74, 86, 88, 95] and VE [44, 76, 79, 87, 90, 93, 94, 96] approaches, and sub-acute [44, 74, 76, 86] and chronic [79, 87, 88, 90, 93–96] populations were represented (see Fig. 6). There was no significant difference in treatment effect [*Q*(2) =0.35, *p* = 0.72] between the four to six week follow-up (*g* = 0.36, *p* = 0.02), the eight to 26 week follow-up (*g* = 0.58, *p* < 0.01), and the overall effect of VR observed immediately following intervention (*g* = 0.46, *p* < 0.01). Differences between CG and VE approaches were not statistically significant at either follow-up [4–6 weeks: *Q*(1) = 2.03, *p* = 0.15; 8–26 weeks: *Q*(1) = 0.10, *p* = 0.76]. Overall, *small* to *medium* effects for both *Body Structure/Function* and *Activity* level outcomes were observed at both the four to six week and the eight to 26 week follow-ups. Only three studies examined *Participation* outcomes at a follow-up [44, 87, 90], which were *small*, and non-significant (*p* = 0.48), in keeping with the pre-post findings. No studies examined cognitive outcomes at follow-up. Consistent with the pre-post data analysis, treatment effects did not vary as a function of the implementation parameters (i.e. dose, daily intensity, weekly intensity, frequency, duration), or recovery stage (i.e. sub-acute vs. chronic).

## Discussion

VR is an engaging form of therapy for stroke [19] and suggested to enhance motor, functional, and cognitive performance [11, 19, 46, 54], whether delivered via VE [11, 46] or CG [11]. While recent reviews of VR therapy have shown improvement in upper limb function, superior to conventional physical therapy [11, 19, 46, 58, 62], we know little of treatment effects across all ICF-WHO levels and how outcomes vary along different implementation parameters and design factors [19, 46], resulting in uncertainties about the optimal training protocol that affords the greatest efficacy. The aim of this study was to address these gaps in understanding by analyzing the current evidence base on VR of upper-limb and cognitive function in stroke, in a combined systematic review and meta-analysis.

Overall, the current review of 33 RCTs found that when compared with conventional therapies, VR interventions produced a *small* to *medium* overall effect (*g* = 0.46), above and beyond conventional physical rehabilitation. Specifically, s*mall* to *medium* effects were observed on *Body Structure/Function* (*g* = 0.41) and *Activity* outcomes (*g* = 0.47), while *Participation* outcomes (*g* = 0.38) were highly variable (*I*^2^ = 65%) but overall non-significant (*p* = 0.26). A *small* to *medium* effect on cognitive outcomes was shown (*g* = 0.41), albeit based on only four studies. Intriguingly, the effect of VR was not moderated by dose-related parameters, and no moderator effects for chronicity were evident. These results are discussed in detail below.

### Overall effectiveness of virtual rehabilitation

The extent of motor recovery after conventional stroke rehabilitation is often “modest” [100, 101] with no significant advantage between different approaches [100, 102]. When compared with these conventional interventions (including occupational therapy and physiotherapy), the current meta-analysis showed an additional *small* to *medium* treatment effect in favor of VR, above and beyond the gains of treatment as usual. The magnitude of this benefit was comparable to that shown in earlier quantitative reviews [11, 19, 46, 65] and reflects an important advance in rehabilitation outcomes. Other attempts to identify novel adjunctive therapies to boost the effects of conventional rehabilitation have been less successful. For example, a review of robotic-assisted therapy for stroke patients with upper-limb impairment [103] showed no significant difference between intensive conventional therapy and robotic-assisted therapy groups in terms of motor recovery, activities of daily living, strength, and motor control.

### Virtual environment versus commercial gaming systems

The current review evaluated two main types of VR interventions: purpose-designed VE platforms were examined in 19 studies and commercially available CG systems in 14. Previous reviews have also examined the separate impact of these two types of intervention, but with too few CG studies to make any firm conclusions about relative efficacy [11, 46]. In contrast to the previous two major reviews, which included only 17% [11] and 22% [46] CG-based studies, almost half (42%) of the studies included in the current review were CG-based interventions, suggesting a growing interest in off-the-shelf solutions.

In the current review, both VE and CG intervention types were significantly superior to conventional therapies, with *medium* effect sizes observed for VE platforms (*g* = 0.59) and *small* effects for CG systems (*g* = 0.33). This difference between VR approaches was statistically significant, and suggests that while both VE and CG systems afford good training effects overall, VE-based systems are somewhat superior [46]. This finding supports the value of customizing rehabilitation tasks according to the clinical needs and capacities of patients. Consistent with previous reviews [11, 46] the positive effect of VE approaches was observed mainly on outcomes at the *Body Structure/Function* and *Activity* levels of the ICF, which is discussed in the next section.

### Virtual rehabilitation outcomes by domains of function

Over 50 different outcome measures were used by studies included in the current review, underlining the importance of standardized classification using the ICF-WHO [58, 104]. For outcomes at the *Body Structure/Function* and *Activity* levels, effects sizes for VR (VE and CG combined) were significant (0.41 and 0.47, respectively). Effects at these levels of the ICF-WHO were more pronounced, however, when VE systems were considered separately: 0.54 and 0.62 for *Body Structure/Function* and *Activity*, respectively, compared with 0.27 and 0.32 for CG systems. The results for VE approaches were comparable to previously reported effect sizes at the *Body Function* (*g*   =   0.48), and *Activity* (*g*   =  0.54) levels [11], which had been based on outcomes from both upper and lower limb interventions combined.

The current meta-analysis of RCTs (published up to June 2017) showed strong evidence of meaningful change across the *Body Structure/Function* and *Activity* levels of the ICF-WHO, unlike earlier reviews [46, 58]. First, we showed significant effects at the *Body Structure/Function* level, where the earlier review of Laver and colleagues showed no change on a group of “other outcomes” that were largely at this ICF-WHO level. Second, our review showed that the largest effect sizes were consistently identified at the *Activity* level whereas Palma and colleagues [58] found inconclusive support and Laver et al. [46] reported relatively small effects on upper-limb function (*d* = 0.28). Finally, treatment effects at the *Participation* level were *small* (*g* = 0.38) and non-significant. Variation in the magnitude of effect across studies in our review (*g* ranging from − 0.37 to 2.04 over five studies) may reflect issues in the assessment of participation outcomes, which is currently an imprecise science [105].

### Cognitive outcomes

While cognitive impairment is common post-stroke [16, 20, 27], and cognitive and motor systems overlap at a structural and functional level [9, 20], only four [53, 54, 91, 97] studies included in the current meta-analysis measured cognitive outcomes. While preliminary, the overall effect of VR on cognition was encouraging, with a mean effect size of *g* = 0.45. The limited number of studies did not permit any conclusions about the superiority of either VE or CG approaches. Palma and colleagues [58] also reviewed cognitive outcomes (from four RCTs), but found no advantage for a VR approach. However, the relevance of several included studies was questionable. One study compared VR with a computerized cognitive rehabilitation program, not with physical therapy [106], a second study contained no identifiable cognitive outcome measures [107] and in a third study, the mental function under investigation was mood state, rather than cognitive status [75]. The fourth study was also included in the current meta-analysis [91]. The results of the current review appear more valid, and provide encouragement that VR can contribute to cognitive rehabilitation. Moving forward, researchers and clinicians are encouraged to be mindful of the inter-relationship between motor and cognitive systems [9] and the potential cognitive benefits of motor-based stroke rehabilitation using VR [25, 31, 54]. For example, a within-group study by Kizony et al. [51] found preliminary evidence supporting the interaction between motor and cognitive function in stroke patients undergoing VR. A more recent study by Subramanian and colleagues [50] provided further evidence of the association between cognitive and motor recovery. Moreover, it was shown that patient’s psychological well-being can also affect motor learning using VR [50], and should also be taken into account in future studies of VR in stroke.

### Implementation parameters and design factors

*Dose-effect relationships* remain inconclusive in the VR area, and in need of further investigation. Reviewing the literature published between 1999 and 2004, Crosbie and colleagues [60] found VR was most commonly delivered three times per week for 1–1.5 h, over a 2–4 week period (i.e. 6–18 h total). Similarly, in their review of the literature from 2008 to 2015, Palma et al. [58] reported the average dose of VR was 17.6 h for upper limb motor function rehabilitation, and 13.2 h for motor activity rehabilitation. These trends were continued in the current review, with the average VR intervention comprised of 40 min sessions delivered three days per week for 6 weeks, for a total of approximately 12 h. However, there was large variability in these implementation parameters, with protocols providing up to 60 min sessions, up to five times per week, for as many as 36 sessions. While a higher number of repetitions and longer training times are argued to be more beneficial for motor learning [108], VR outcomes are argued not to be exclusively dependent on dose [46]. In the current review, moderator analysis also found no clear added benefit of higher doses or massed practice of VR, suggesting a ceiling after which gains plateau. While the dose of rehabilitation may not be the most important factor affecting recovery [109], the average intensity, frequency and duration of VR training identified in the current review appeared to provide an effective schedule for cognitive and motor function outcomes, while reducing the chance of participant fatigue or burn out that may occur under higher intensity training.

#### Active versus passive control groups

There was no difference in effect sizes (*g* = 0.45 c.f. 0.48) for interventions that utilized an *active* control group (i.e. additional conventional therapy beyond just treatment as usual) or a *passive* one (i.e. treatment as usual only). This was an unexpected finding as active control group designs are preferred for their capability to presumably control for Hawthorne effects and other biases when comparison groups are not balanced in terms of time in therapy. However, the current findings suggest that the use of a passive control group does not inflate the effect size for the intervention group. It also suggests that those treatment strategies embedded in active conditions may not add substantially to the training effects usually observed for treatment as usual. This finding provides credence to studies that lack the resourcing to implement an active control group design and just proceed with a treatment as usual group, which is the case more often than not in rehabilitation research [110].

#### Sub-acute versus chronic stage

Moderator analysis showed that VR administered in the sub-acute (*g* = 0.25) and the chronic stages (*g* = 0.51) were both effective. However, only seven studies included in the current review intervened early after stroke, and the optimal time window for delivering VR remains an issue for further study. For the chronic group, there was a large variation in the time since stroke (range 6 months to several years). While it may be argued that participants with longer-term impairment remained responsive to VR treatment, early intervention is still recommended [111, 112] to address neurological changes before chronic disability ensues [101]. As particular treatment modalities are refined with advances in the technology (e.g., delivery of augmented feedback) there will be unique opportunities to enhance neuroplastic changes during this critical time period [113].

### Outcomes at follow-up

A third of all studies included follow-up assessment [44, 74, 76, 79, 86–88, 90, 93–96]. Participant retention was generally high, with only one study experiencing attrition rates over 10% at follow-up [86]. Follow-up duration was four weeks in five studies [44, 76, 79, 86, 94], six weeks in one study [96], eight weeks in two studies [90, 93], 13 weeks in three studies [74, 88, 95] and 26 weeks in one study [87]. Over all follow-up durations, the initial gains reported immediately following VR training were preserved. These findings are encouraging, and suggest that a discrete period of VR can affect longer-lasting improvements in overall motor function, and on ICF-WHO *Body Structure/Function* and *Activity* level outcomes in particular. By comparison, there is accumulating evidence that early improvements after conventional rehabilitation may not be sustained long-term after stroke [114, 115]. Notably, the current review showed that gains were maintained regardless of VR approach (CG or VE), dosing (i.e. frequency, intensity, or duration of training), or stage of recovery (i.e. sub-acute or chronic). Surprisingly, no studies examined cognitive outcomes at follow-up, and the durability of post-training improvements in this domain remains unknown. The stability of gains over periods longer than six months has also not been explored but should be encouraged in future research. Also for further study are questions about whether booster sessions or other strategies such as activity monitoring, goal setting, or feedback systems [116] are needed to optimize stroke survivors’ longer-term outcomes after VR.

### Risk of bias

To maximize the quality of evidence in this review, all of the included studies were Level 1b (RCTs) to Level 2b (small RCTs) according to the Centre for Evidence-Based Medicine [117]. As evaluated formally using the PEDro Scale, the quality of studies was also generally *high*. Not surprisingly, the only design component consistently omitted was the blinding of participants, which is difficult to achieve using novel and distinct interventions like VR [46]. One study described their methodology as a double-blind procedure [82], but while participants may have been naïve to the intended outcomes of the study it is unlikely they were unaware of their group assignment (VR vs. passive control group). The current study did not include a search and review of unpublished (grey) literature, which could be important to account for publication bias (or file drawer effect) [118]. The current review specifically focused on published, peer-review articles to ensure the high quality of included data, but performed a fail-safe *N* [119] calculation to account for missing studies and grey literature. Fail-safe *N* value of 439: that is, 13 missing studies for every observed study would be required for the overall effect of VR to be nullified, further supporting the observed efficacy of VR. With the risk of biases minimized, we are confident that VR, and in particular VE, can be recommended as a useful adjunct or alternative to conventional therapy when retraining motor and cognitive function following stroke. The ability of VR to enhance experience-dependent neuroplasticity is suggested but demands new research to investigate changes at the brain level. These recommendations are discussed below.

### Limitations and directions for future research

The current review did not extend to a formal investigation of *active ingredients* (i.e. those aspects of VR that are having the most profound impact on functioning), which remains an important and unresolved issue in VR. What makes this issue particularly hard to dissect is the sheer variety in types of interface, augmented feedback, setting, and so on across different studies. It is likely there are both generic and more specific effects of VR on neuroplastic changes and the process of skill learning itself. For example, novelty and engagement are critical to any rehabilitation paradigm and can be captured by a number of well-designed (game-like) VE platforms, or popular CG systems like Wii, Kinect, and PlayStation [90]. The capability of VR to scale levels of difficulty and to provide appropriate rewards to users in the context of gameplay and advancement between levels is critical to CG. Use of augmented feedback (known to be important in motor learning) is one factor that will vary greatly with interface design and the type of human-computer interaction that a given system affords [36]. Componential approaches to system evaluation will be particularly valuable in future research, varying a critical ingredient that is thought to predict an outcome while holding all other factors constant.

The effect of different neurological characteristics on VR rehabilitation outcomes is also in need of examination. Most studies in the current review used mixed samples of hemorrhagic and ischemic clients; only three studies sampled exclusively ischemic stroke patients [78, 86, 93]. Some studies suggest that hemorrhagic stroke may result in more severe cognitive, motor and functional impairment than ischemic stroke [120, 121]. By comparison, other work shows that differences between stroke types are marginal across these domains [122–124]. Future investigations would benefit from comparison of these stroke types to test the impact of mixed cohorts. Moreover, consistent reporting of details including lesion location (e.g. Oxfordshire Community Stroke Project Classification) and hemisphere, and initial severity and symptom profiles (e.g. National Institutes of Health Stroke Scale; modified Rankin Scale), will assist in identifying the neurological characteristics of stroke more or less responsive to VR.

Many studies in the current review had small participant numbers. With only an average of 15 participants per group, a number of studies lacked sufficient statistical power to examine more than one or two outcomes [125], and were likely underpowered to examine interactions, predictors, or multivariate effects. As we recommend examining outcomes across all three levels of the ICF-WHO, including cognitive outcomes, larger-*n* studies are recommended in the future, with power calculations pointing to in excess of 20 participants per treatment arm.

Variation in the choice of primary outcome measure also limits comparison between studies. The VR research field should consider developing a consensus statement on evaluation research to aid the consistency in measurement. For example, at the *Body Structure/Function* level, the FMA might be considered as a “gold standard” in the absence of a better tool at this point in time. At the *Activity* level, the Box and Blocks Test has been shown to correlate very highly with longer test batteries that assess skill (like the Action Research Arm Test), and could be included as a standard, easy to administer measure. Due to a limited number of studies reporting cognitive outcomes, the current review could only report on cognition as a unified concept, rather than its more specific domains. Taken together, there is a need to include well-validated assessment of cognition.

The far transfer of training effects to important aspects of daily functioning, independence, and quality of life was examined in only five of the current studies, all of which utilized a VE approach [13, 44, 57, 87, 90]. The overall effect size on *Participation* outcomes was non-significant (*p* = 0.26). This result mirrored an earlier review by Saposnik and Levin [19] which identified only one study reporting on (social) participation. By comparison, the review of Lohse and colleagues [11] identified a single study that reported a significant effect on participation outcomes. However, Lohse and colleagues [11] misclassified the Jebsen Taylor Hand Function Test as a *Participation* measure when in fact it is usually classified as an *Activity* level outcome [59, 126]. In our review we observed high variability in results, ranging from non-significance (*g* = − 0.37) to a *large* significant effect (*g* = 2.04). The latter study was the only home-based intervention and involved a control group that completed their conventional rehabilitation before the study started. One or both of these unique features may explain the size of the observed effect. Overall, efficacy of VR at the *Participation* level of the ICF-WHO remains inconclusive (see also [58]) and the amount of evidence bearing on it is very limited [19, 58]. We recommend examination of far-transfer *Participation* outcomes as standard practice.

### Implications for practice

Knowledge of the pattern of treatment effects across ICF levels has important implications for the design of tailored interventions for stroke and evidence-based recommendations for care. Stronger effects for VE-based systems over CG suggest that the added expense of acquiring purpose-designed systems might be a good investment for clinicians, backed, of course, by well-controlled evaluation studies. However, at this point, we still do not have sufficient data to make strong predictions about the (far-transfer) effects of such training on *Participation*. In cases where cost and access to VE systems is an issue, CG systems will still leverage outcomes at the *Body Structure/Function* and *Activity* levels.

There is too little data to yet make firm conclusions about the impact of VR on cognition. However, there are a number of examples where cognitive performance has been enhanced through what are essentially motor-based interventions for the upper limbs. For example, for patients with traumatic brain injury (TBI), Mumford and colleagues [38] showed VR produces a significant subjective improvement in attention and memory function.

Taken together, clinicians and researchers alike are encouraged to seek out purpose-designed VE systems that can boast high-quality evidence for their efficacy. The principled and evidence-based approach to the design, implementation, and evaluation of VE instruments confers a considerable therapeutic advantage at the level of functional movement skill. As a matter of course, future research needs to extend the evaluation of outcomes across all ICF-WHO levels.

As mentioned, the moderator analysis was unable to detect a linear dose-response relationship, as no advantage for higher dosing on any of the VR approaches or rehabilitation outcomes were found. Future studies should seek to explore the more active ingredients of VR, to maximize both the efficacy and the efficiency of treatment rather than simply relying on higher doses. Implications for patient engagement, retention, and satisfaction remain to be explored.

## Conclusion

The physical and cognitive impairment resulting from stroke is persistent and prominent, and the prospect of recovery both compelling and elusive. VR interventions offer the unique opportunity for patients to interact in an enriched environment, providing structured, scalable training opportunities augmented by multi-sensory feedback to enhance skill learning and neuroplasticity through repeated practice. Findings from this review suggest VR has an added advantage over conventional interventions, and can produce immediate and longer-term improvements in motor function and the performance of cognitive and motor activities following stroke. The evidence-based efficacy of a VR approach extends to patients in both the acute and chronic recovery stage, utilizing a spaced training schedule delivered via either purpose-designed or commercially available systems. Continued application of this promising technology is encouraged, to refine our understanding of the factors contributing to the beneficial effects of VR, and to promote the transfer of gains to participation outcomes.

### Additional files

Additional file 1: **Figure S1.** Forest plot showing the overall main effect-sizes of Virtual rehabilitation after stroke for each individual study using the random-effects model. (TIFF 1240 kb)

Additional file 2: **Figure S2.** Forest plot showing the overall main effect-sizes for each individual study of virtual rehabilitation on the International Classification of Functioning and cognitive outcomes after stroke using the random-effects model. (TIFF 760 kb)

## Appendix

**Table 6:** Sample search strategy for the Ovid MEDLINE database

Set #	Search String
1	Cerebral Ischemia/ or exp. Cerebrovascular Accidents/ or exp Ischemia/ or exp Cardiovascular Disorders/ or Stroke.mp.
2	Cerebrovascular disease/ or exp basal ganglion hemorrhage/ or exp brain hematoma/ or exp brain hemorrhage/ or exp brain infarction/ or exp brain ischemia/ or exp carotid artery disease/ or cerebral artery disease/ or exp cerebrovascular malformation/ or exp intracranial aneurysm/ or exp occlusive cerebrovascular disease/ or stroke/ or stroke unit/ or stroke patient/
3	(Hemorrhag* or Subarachnoid Hemorrhage or intracranial hemorrhage).mp.
4	(Poststroke or post-stroke or cerebrovasc* or cerebral vascular or brain vasc* or cerebral vasc* or cva* or apoplexy* or SAH).mp.
5	(Brain* or cerebr* or cerebell* or intracran* or intracerebral N5 isch?emi* or infarct* or thrombo* or emboli* or occlus*).mp.
6	1 or 2 or 3 or 4 or 5 or 6
7	(Virtual real* or virtual-real* or VR).mp.
8	(Augment* reality or virtual reality or augment* gam* or virtual gam*).mp.
9	(Video gam* or computer gam* or gaming consol* or interactive gam* or Nintendo Wii or kinect or nintendo or playstation or xbox or gam* program).mp.
10	(Virtual adj3 (environment* or object* or world* or treatment* or system* or program* or rehabilitation* or therap*)).mp.
11	8 or 9 or 10 or 11
12	6 and 11

## Abbreviations

ADLs: Activities of Daily Living
BBT: Box and Blocks Test
CG: Computerized Gaming
CT: Conventional Treatment
FMA: Fugl-Meyer Assessment
ICF-WHO: The World Health Orgainzation’s International Classification of Functioning, Disability, and Health
PEDro: Physiotherapy Evidence Database
RCT: Randomized Controlled Trial
VE: Virtual Environments
VR: Virtual Rehabilitation
