# Supplementary figures and images for: What do randomized controlled trials say about virtual rehabilitation in stroke? A systematic literature review and meta-analysis of upper-limb and cognitive outcomes
Source: J Neuroeng Rehabil. 2018 Mar 27;15:29. doi: 10.1186/s12984-018-0370-2 (PMC5870176; doi:10.1186/s12984-018-0370-2)

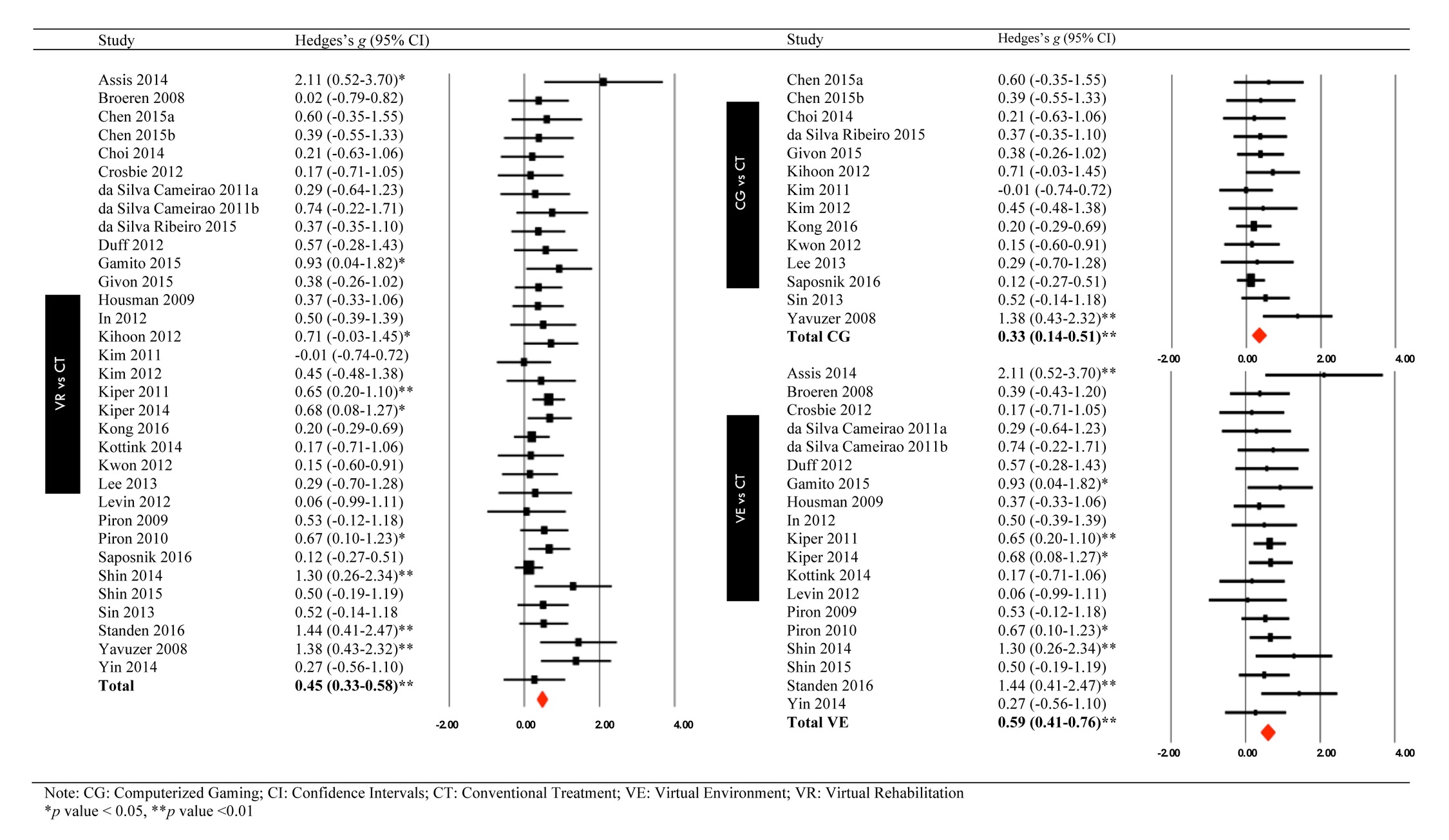

Supplement: Supplementary file 1 — Figure S1. Forest plot showing the overall main effect-sizes of Virtual rehabilitation after stroke for each individual study using the random-effects model. (TIFF 1240 kb) [file 12984_2018_370_MOESM1_ESM.tif]

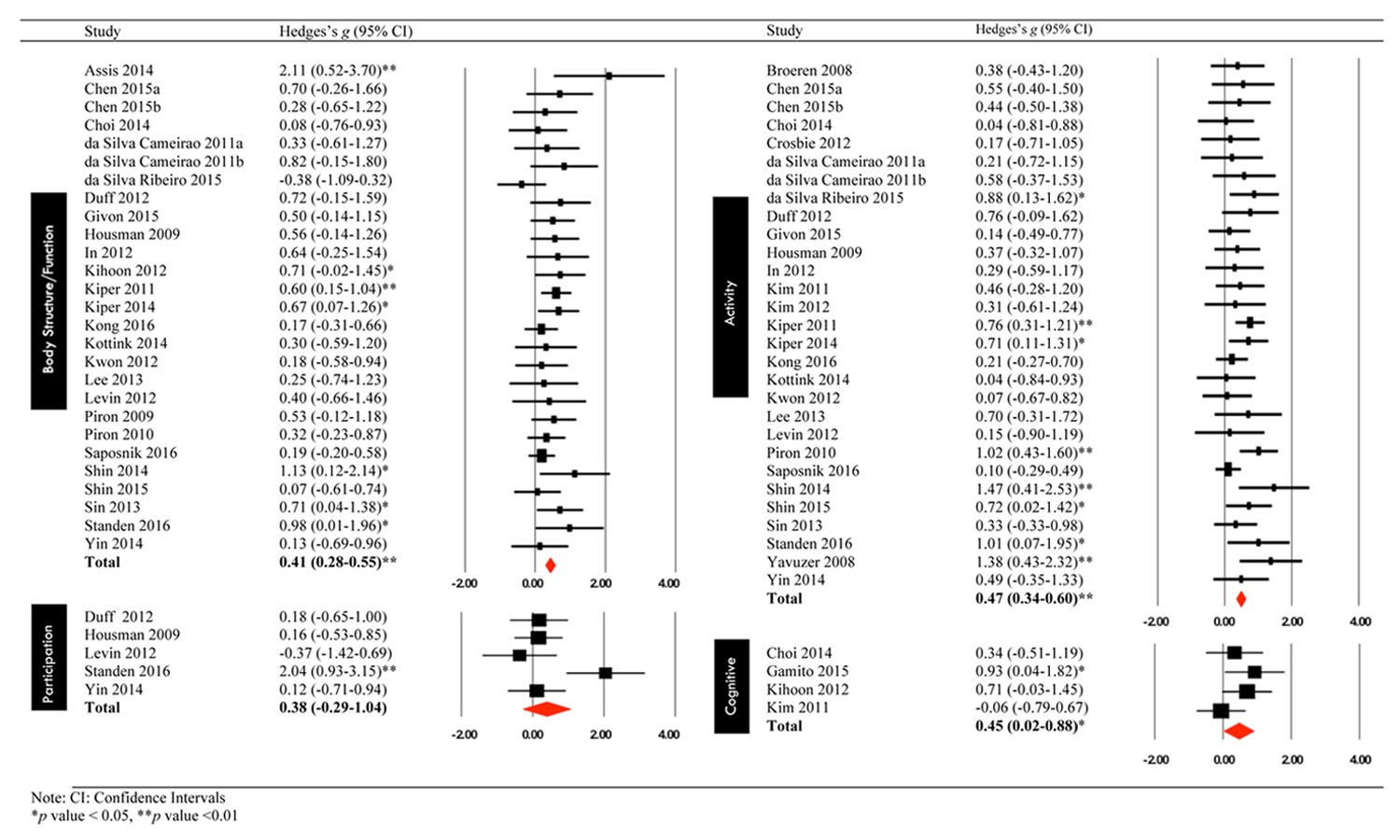

Supplement: Supplementary file 2 — Figure S2. Forest plot showing the overall main effect-sizes for each individual study of virtual rehabilitation on the International Classification of Functioning and cognitive outcomes after stroke using the random-effects model. (TIFF 760 kb) [file 12984_2018_370_MOESM2_ESM.tif]
